# Assessing Methotrexate Adherence in Rheumatoid Arthritis: A Cross-Sectional Survey

**DOI:** 10.1007/s40744-015-0011-1

**Published:** 2015-05-13

**Authors:** Dana B. DiBenedetti, Xiaolei Zhou, Maria Reynolds, Sarika Ogale, Jennie H. Best

**Affiliations:** 1 RTI Health Solutions, Research Triangle Park, NC USA; 2 Genentech, Inc., South San Francisco, CA USA

**Keywords:** Cross-sectional survey, Medication adherence, Methotrexate, Rheumatoid arthritis

## Abstract

**Introduction:**

Limited data are available to explain nonadherence to methotrexate (MTX) therapy in patients with rheumatoid arthritis (RA). Better understanding of patterns of MTX use and reasons for nonadherence may help identify patients who would benefit from alternative RA treatments and potentially aid in developing strategies to increase overall adherence. The purpose of this study was to assess patients’ self-reported adherence to MTX and to identify reasons for nonadherence.

**Methods:**

Patient panel members in the US self-reporting a diagnosis of RA of ≥3 months’ and current MTX use of ≥4 weeks’ duration, with or without concomitant use of another RA prescription medication, participated in this cross-sectional, web-based survey.

**Results:**

The sample population (251 MTX monotherapy, 250 MTX combination therapy) was predominantly female, white, non-Hispanic, and educated; 48% were 18–44 years-old, 47% had medical comorbidities, 66% were first diagnosed with RA ≤5 years earlier, 51% reported MTX use of <1 year, and 83% reported oral MTX use. Forty-two percent reported not taking MTX exactly as prescribed. Reasons for nonadherence included forgetting to take it (33%), not needing it when feeling well (24%), and concern about long-term safety (24%). Among nonadherent patients, 53% took smaller doses, 52% skipped doses, and 6% reported other nonprescribed ways of taking MTX. Younger age, male sex, and shorter duration of MTX use were associated with poorer self-reported adherence. Compared with monotherapy patients, combination therapy patients, particularly those taking ≥2 other RA prescriptions, were less likely to report high adherence.

**Conclusion:**

Nearly half the sample reported poor MTX adherence because they forgot to take it, thought it was not needed when they felt well, or had long-term safety concerns. Patients taking ≥2 other RA prescription medications were less likely to report good adherence. Reducing treatment burden without sacrificing efficacy may be a strategy worth evaluating.

**Electronic supplementary material:**

The online version of this article (doi:10.1007/s40744-015-0011-1) contains supplementary material, which is available to authorized users.

## Introduction

Rheumatoid arthritis (RA) is a chronic autoimmune disease characterized by inflammation of the membrane lining the joints (the synovium). Patients with RA may experience pain, redness, swelling, stiffness, and restricted movement around the joints of the hands, feet, elbows, knees, and neck that lead to loss of function, deformity, and disability.

Approximately 1.5 million people in the US, or 0.6% of the population, have RA [1]. RA occurs 2–3 times more frequently in women than in men [2]. Although RA can affect people at any age, including children, onset usually occurs between 40 and 60 years of age [3].

Current treatment options include medication, reduction of joint stress, occupational and physical therapy, and, in some cases, surgery. Pharmacologic treatments for RA generally fall into one of the three drug categories: nonsteroidal anti-inflammatory drugs (NSAIDs), corticosteroids, and disease-modifying antirheumatic drugs (DMARDs). Although the first two classes generally have a short onset of action, clinical response with DMARDs can take weeks to months [4]. Rheumatologists often use short courses of corticosteroids for acute flares of RA or as a bridge between other DMARDS, but the use of these drugs in RA is controversial [5]. Although both NSAIDs and DMARDs help alleviate active RA symptoms, only DMARDs alter the progression of RA.

Worldwide, methotrexate (MTX) is a common first-line DMARD treatment for patients with RA. Early initiation of MTX in patients with RA helps manage joint destruction and slows disease progression [6], and most patients who are prescribed MTX are still taking it after 5 years [7]. In patients with partial responses to MTX, additional treatments are often added. MTX can be combined safely with almost all other DMARDs approved for RA [4].

Although the long-term efficacy, tolerability, and safety of MTX have been well documented [6], the clinical response to MTX treatment and the frequency of adverse events from the drug may impact patient adherence. Patient adherence can be influenced by duration of disease, disease activity, and medical comorbidities [8]. Better understanding of patterns of MTX use (as monotherapy and in combination therapy), patient and disease characteristics, adherence, and reasons for nonadherence may help in identifying patients who would benefit from alternative RA treatments and in devising strategies to increase overall patient adherence. Thus, the primary objectives of this study were to assess patients’ self-reported adherence to MTX and to identify reasons for nonadherence.

## Methods

### Study Design

The current study was a cross-sectional, web-based survey in the US of patients self-reporting a clinician-administered diagnosis of RA and current treatment with MTX, with or without concomitant use of another prescription medication for RA. Participants were recruited from an existing opt-in online panel of patients with RA in the US developed and maintained by All Global [9], a leading data collection agency focused solely on health care research that specializes in hard-to-reach populations and maintains access to persons who have been prescreened for specific characteristics or medical conditions, such as RA. Respondents were selected from the panel at random. In the current study, once panel members logged into their all global accounts, they were directed to the survey to complete a few screening questions. Participants meeting eligibility criteria then reviewed and electronically provided informed consent before completing the online questionnaire. This research was conducted in compliance with the Declaration of Helsinki. All eligible respondents received $2 for completing the survey. The current study was fielded from September 27, 2013 to October 9, 2013 following RTI International’s ethics committee approval.

The web-based questionnaire was self-administered. Participants were encouraged to complete the questionnaire in a timely manner. Once they started the questionnaire, they were able to stop at any point and then resume it from that point. Participants who completed the questionnaire were not allowed to access the survey again.

### Study Population

The survey participants were adults who met all the following criteria: self-reported physician diagnosis of RA, diagnosis of RA for at least 3 months, current use of MTX for at least 4 weeks, and ability to read and understand English and provide informed consent.

Eligible participants were classified into one of two groups based on their current MTX use: MTX monotherapy, currently taking MTX without any other prescription medications for RA; MTX combination therapy, currently taking MTX and at least one other prescription medication for RA. The target sample size for survey completion was 500 participants (250 per MTX group).

### Questionnaire Content

The survey questionnaire assessed the following areas:

- demographic characteristics and health insurance information;
- current use of MTX and other prescription medications for RA;
- formulation, frequency of use, and dose of MTX;
- MTX adherence and reasons for nonadherence;
- satisfaction with MTX treatment;
- general health, including as assessed by the Modified Health Assessment Questionnaire (MHAQ). The eight-item MHAQ [10, 11] assesses disease-related disability, discomfort, and quality of life in patients with RA. Each item is scored from 0 (without any difficulty) to 3 (unable to do). The total score is the sum of the scores from the 8 items divided by 8. The total MHAQ score ranges between 0.0 and 3.0, with higher scores indicating greater overall disability. MHAQ scores <0.3 are considered normal, though the average MHAQ score in the general population increases with the participant’s age.

With the exception of the MHAQ, all questionnaire items were developed specifically for this study to address study-related objectives. Completion time was approximately 10 min.

### Data Analysis

Data analysis was primarily descriptive and included tables summarizing the demographic and clinical characteristics of the sample. In addition, participants’ responses to each of the survey questions, as well as the derived MHAQ total score, were summarized by MTX group (monotherapy or combination therapy) and for the overall sample. Descriptive statistics (e.g., mean, standard deviation, range) for continuous variables and frequencies with percentages for categorical variables are reported. For each question, missing data (<1%) were excluded from analysis, and no imputation was performed.

Patient adherence was assessed by the question, “During the past 4 weeks, how much of the time did you take your methotrexate exactly as prescribed by your doctor?” Patients were able to select “none of the time,” “a little of the time,” “some of the time,” “most of the time,” or “all of the time.”Patients who selected “most” or “all” of the time were classified as showing high adherence, and those who selected “none,” “a little,” or “some” of the time were classified as showing low adherence. A logistic regression model was also used to identify factors associated with level of adherence to MTX. The backward selection method was used to eliminate nonsignificant variables (*P* > 0.05). The full model included age, sex, level of education, race/ethnicity, type of health insurance, prescription drug coverage, duration of RA, duration of MTX use, dose of MTX, satisfaction with MTX, MHAQ total score, number of other prescription medications for RA, and number of medical comorbidities. Analyses were performed using SAS software, Version 9.3 (SAS Institute Inc., Cary, NC, USA).

## Results

**Fig. 1:**
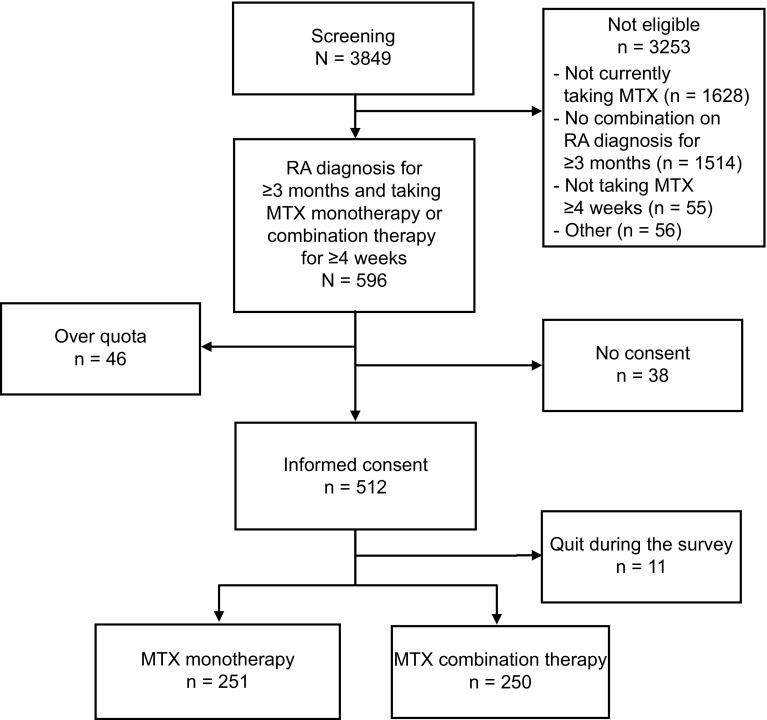
Study enrollment. Initially, 596 patients met initial eligibility criteria. However, only 550 patients (512 + 38) were asked for consent. The remaining 46 patients were not asked for consent because the target sample size for that treatment group (monotherapy or combination therapy) had been reached (quota met). Before group quotas were met, 93% (512/550) of the patients provided informed consent. *MTX* methotrexate, *RA* rheumatoid arthritis

The screening portion of the survey questionnaire was completed by 3,849 RA panelists. In total, 596 participants (15%) met all eligibility criteria and were classified into 1 of the MTX groups. Of the 512 eligible participants who provided informed consent, 501 completed the questionnaire (251 in the MTX monotherapy group, 250 in the MTX combination therapy group; Fig. 1). Figure 1 depicts the passage of participants from screening to enrollment.

### Demographics and Medical Comorbidities

Overall, study participants were predominantly female (62%), white (71%), and non-Hispanic (84%); 79% reported at least some college education. Nearly half the study participants (48%) were 18–44 years of age, and 30% were 45–54 years of age (Table 1). Nearly half (47%) reported 1 or more of the medical comorbidities assessed (Table 2), and nearly one quarter (23%) reported diabetes; 22% reported a MHAQ total score <0.3, which suggests no disability.

**Table 1:** Demographic characteristics by MTX treatment group

Characteristic, *n* (%)	MTX monotherapy *n* = 251	MTX combination therapy *n* = 250	Overall *n* = 501
Age, years			
18–35	78 (31)	50 (20)	128 (26)
36–44	61 (24)	51 (20)	112 (22)
45–54	67 (27)	81 (32)	148 (30)
55–64	44 (18)	63 (25)	107 (21)
65–75	0	5 (2)	5 (1)
76 and older	1 (<1)	0	1 (<1)
Sex			
Male	108 (43)	84 (34)	192 (38)
Female	143 (57)	166 (66)	309 (62)
Education			
Less than high school graduate	4 (2)	2 (1)	6 (1)
High school graduate	53 (21)	48 (19)	101 (20)
Some college, technical training/licensure	69 (27)	87 (35)	156 (31)
College graduate	97 (39)	73 (29)	170 (34)
Advanced or professional degree	28 (11)	40 (16)	68 (14)
Hispanic, Latino, or Spanish origin or descent			
Yes	31 (12)	50 (20)	81 (16)
No	220 (88)	200 (80)	420 (84)
Race			
White	175 (70)	180 (72)	355 (71)
Black/African American	43 (17)	44 (18)	87 (17)
American Indian or Alaska native	3 (1)	6 (2)	9 (2)
Asian	18 (7)	9 (4)	27 (5)
Native Hawaiian or other Pacific Islander	1 (<1)	0	1 (<1)
Other	11 (4)	10 (4)	21 (4)
No answer	0	1	1
Other medications for RA			
Prednisone	–	82 (33)	–
Adalimumab (Humira)	–	66 (26)	–
Hydroxychloroquine (Plaquenil)	–	52 (21)	–
Etanercept (Enbrel)	–	51 (20)	–
Infliximab (Remicade)	–	29 (12)	–
Azathioprine (Azasan, Imuran)	–	24 (10)	–
Tocilizumab (Actemra)	–	23 (9)	–
Certolizumab pegol (Cimzia)	–	23 (9)	–
Sulfasalazine (Azulfidine)	–	20 (8)	–
Leflunomide (Arava)	–	18 (7)	–
Abatacept (Orencia)	–	16 (6)	–
Tofacitinib (Xeljanz)	–	15 (6)	–
Anakinra (Kineret)	–	14 (6)	–
Rituximab (Rituxan)	–	13 (5)	–
Other	–	11 (4)	–
Golimumab (Simponi)	–	7 (3)	–

*MTX* methotrexate, *RA* rheumatoid arthritis

The sum of the percentages exceeds 100% because respondents were allowed to select more than 1 response

**Table 2:** MHAQ by MTX treatment group

Characteristic	MTX monotherapy *n* = 251	MTX combination therapy *n* = 250	Overall *n* = 501
Diagnosed medical comorbidities, *n* (%)^a^			
Heart disease	17 (7)	30 (12)	47 (9)
Diabetes	47 (19)	66 (26)	113 (23)
Chronic pulmonary disease	13 (5)	21 (8)	34 (7)
Ulcer	22 (9)	33 (13)	55 (11)
Liver disease	9 (4)	20 (8)	29 (6)
Skin cancer or melanoma	6 (2)	19 (8)	25 (5)
Other autoimmune disease	16 (6)	36 (14)	52 (10)
Other types of cancer	4 (2)	12 (5)	16 (3)
None of the above	156 (62)	108 (43)	264 (53)
MHAQ total score^b^			
Mean (SD)	0.8 (0.5)	0.8 (0.5)	0.8 (0.5)
<0.3, *n* (%)	57 (23)	52 (21)	109 (22)

*MHAQ* Modified Health Assessment Questionnaire, *MTX* methotrexate, *SD* standard deviation

^a^The sum of the percentages exceeds 100% because respondents were allowed to select more than 1 response

^b^MHAQ total score <0.3 suggests no disability

Respondents reported taking a mean of 5 (median, 3) unique prescription medications (including MTX) for any condition in a typical week. Of the participants reporting MTX combination therapy (*n* = 250), prednisone was the most commonly reported RA medication used in addition to MTX, followed by adalimumab, hydroxychloroquine, and etanercept. Table 1 presents additional details on the sample’s RA medication use.

### Patterns of Methotrexate Use

Two-thirds of the overall sample (66%) received the diagnosis of RA within the past 5 years, and only 14% received the diagnosis 10 or more years ago. Slightly more than half the sample (51%) reported using MTX for less than 1 year, and 33% reported using it for 1–5 years.

Most respondents (83%) reported using the oral formulation of MTX; no differences in formulation were reported between the MTX monotherapy group (83%) and the combination therapy group (84%). More than half (59%) self-reported that their prescriptions called for them to take MTX orally or to inject it once a week, twice a week (37%), or “other” (5%) (percentages exceed 100% due to rounding).

### Methotrexate Adherence

Forty-two percent of the overall sample indicated that they did not take MTX exactly as prescribed by their doctors at all times during the past 4 weeks (Table 3).

**Table 3:** Adherence to MTX by MTX treatment group

Characteristic, *n* (%)	MTX monotherapy *n* = 251	MTX combination therapy *n* = 250	Overall *n* = 501
Frequency of taking MTX exactly as prescribed			
None of the time	3 (1)	0	3 (1)
A little of the time	21 (8)	26 (10)	47 (9)
Some of the time	26 (10)	28 (11)	54 (11)
Most of the time	59 (24)	48 (19)	107 (21)
All of the time	142 (57)	147 (59)	289 (58)
No answer	0	1	1
Ways in which MTX was taken differently than prescribed^a^			
Total	109	102	211
I took smaller doses than prescribed	57 (52)	54 (53)	111 (53)
I skipped doses	54 (50)	56 (55)	110 (52)
Other	6 (6)	7 (7)	13 (6)
No answer	0	0	0

*MTX* methotrexate

^a^The sum of the percentages exceeds 100% because respondents were allowed to select more than 1 response

**Fig. 2:**
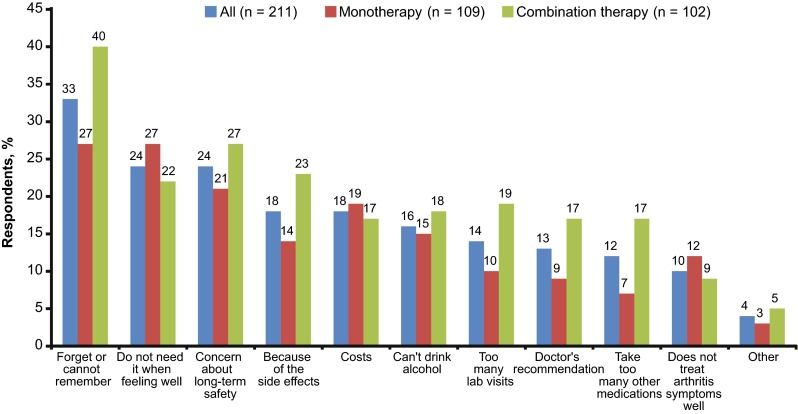
Reasons for nonadherence. Percentages on the bars are based on the sample of patients (*n* = 211) who indicated that they did not always take their methotrexate exactly as prescribed by their doctors

Of the 211 respondents who indicated that they did not always take MTX exactly as prescribed by their doctors, the most commonly reported reasons were forgetting to take it, feeling it was not needed when they felt well, and concern about the long-term safety of MTX (Fig. 2). Forgetting to take MTX as prescribed was more frequently reported in MTX combination therapy (40%) than MTX monotherapy (27%) respondents (Pearson Chi-square test, *P* = 0.0361). Taking “too many other medications” was reported more frequently in the MTX combination group than the MTX monotherapy group (17 vs. 7%; *P* = 0.0362).

Additionally, more than half the 211 respondents who indicated that they did not always take their MTX exactly as prescribed by their doctors reported that they took smaller doses than prescribed (53%) or skipped doses (52%), with similar patterns across MTX groups.

**Fig. 3:**
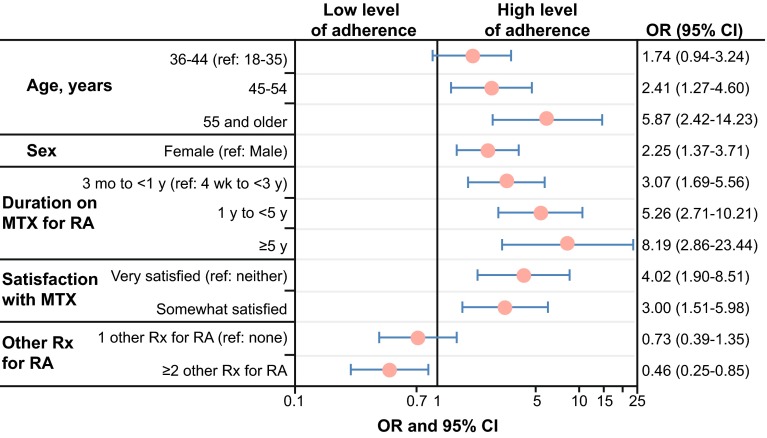
Factors related to adherence level (high [*n* = 396] vs. low [*n* = 104]). Results from the logistic regression analysis with backward selection to remove nonsignificant variables (*P* > 0.05) or for “somewhat” or “very dissatisfied” are not reported because of the small sample size. *CI* confidence interval, *MTX* methotrexate, *OR* odds ratio, *RA* rheumatoid arthritis, *ref* reference, *Rx* therapy

A logistic regression model was used to identify factors associated with level of MTX adherence (most or all of the time vs. none to some of the time). As shown in Fig. 3, results indicated that younger age, male sex, and shorter duration of MTX use were associated with poorer self-reported MTX adherence. Additionally, compared with MTX monotherapy patients, MTX combination therapy patients, particularly those taking ≥2 other prescription medications for RA, were less likely to report “high” adherence (odds ratio [OR], 0.46; 95% confidence interval [CI], 0.25–0.85).

High level of satisfaction was associated with high level of adherence. Compared with the OR in patients who described themselves as “neither satisfied nor dissatisfied,” the OR of high level of adherence was 4.02 (95% CI 1.90–8.51) in patients who were “very satisfied” and 3.00 (95% CI, 1.51–5.98) in patients who were “somewhat satisfied.” Education, race/ethnicity, health insurance, prescription drug coverage, time since RA diagnosis, MTX dose, MHAQ total score, and number of medical comorbidities were not associated with MTX adherence level.

## Discussion

The primary objective of this cross-sectional, web-based survey study was to gain better understanding of adherence to MTX therapy and reasons for nonadherence. Better understanding of the profile of adherence and reasons for nonadherence may help identify strategies that can improve patient adherence and, ultimately, disease management.

In the current study, more than half the survey respondents were relatively “new” MTX users (less than 1 year), and 84% had been using MTX for fewer than 5 years. Although the sample size for those reporting MTX use of 5 years or more was relatively small, a larger percentage of MTX combination therapy than monotherapy patients reported longer-term use (5 years or more). This finding is not surprising given that most patients who are prescribed MTX are still taking it after 5 years, and additional treatments are often added for patients with partial responses to MTX [4]. Patients likely have to be taking MTX for some time with limited success before another medication is added.

Nearly half the patients in the current study reported less than perfect adherence to MTX; similar results were seen across MTX groups. The most frequently reported reasons for nonadherence to MTX included forgetting or not remembering to take it, thinking it was not needed when they felt well, and concern about the long-term safety of MTX. More than half the nonadherent patients reported that they took smaller doses than prescribed, skipped doses, or did both. Patients’ self-reporting of nonadherence is generally not a perfect measure and can be considered a limitation of this study.

More patients reporting that they forgot to take MTX were in the combination therapy group than in the MTX monotherapy group. Forgetting to take MTX as directed may be associated with the fact that combination therapy respondents were slightly older, took more unique medications per week, and reported more comorbidities than MTX monotherapy respondents. Additionally, logistic regression model results indicate that patients with two or more other prescription medications for RA were less likely to report good adherence than those who used MTX monotherapy. Other factors associated with poor adherence include younger age, male sex, and shorter duration of MTX therapy.

Although the current study adds to the existing literature on reasons for MTX nonadherence in patients with RA, several study limitations should be noted. Despite the large sample size used, the overall sample was predominantly female, white, non-Hispanic, educated, insured, and generally not disabled. Thus, it is not known how generalizable these findings are to the broader MTX population, particularly those who are less educated, sicker, and perhaps more disabled or more vulnerable (e.g., those of lower socioeconomic status). Efforts to include these populations in future studies, such as in paper- and/or telephone-administered surveys or recruitment through clinics, may aid in better understanding adherence in these patient populations. Additionally, the participants recruited for this study came from a convenience sample of an existing RA panel who agreed to take health-related surveys as part of their panel participation. Thus, it is possible that, but unknown whether, such panel participants are generally more motivated and more likely to be adherent than nonpanel members. How representative this sample is of the broader RA population is also unknown. Panel participants also self-reported their diagnoses of RA. It is unknown whether any differences in adherence and reasons for nonadherence would be demonstrated in a study in participants with clinician-confirmed diagnoses of RA. Furthermore, there may be additional reasons for MTX nonadherence that were not assessed in the current study (e.g., lack of transportation to obtain it). The most common reasons for nonadherence were generated from the literature and in collaboration with the study sponsor, but it is possible that other reasons factor into patient nonadherence and may be identified in future studies. Finally, the survey questionnaire did not assess more detailed MTX treatment patterns, such as time from initiation of MTX to initiation of another DMARD, length of MTX discontinuation and reasons for restarting MTX, and ways in which specific side effects may affect adherence; these could help elucidate reasons for nonadherence.

This study has several strengths. The first is its large sample size, including both monotherapy and combination therapy patients, which adds to confidence in the findings. Another is that identifying specific reasons for nonadherence and MTX practices in noncompliant patients (e.g., smaller doses, skipping doses) suggests that patient education on proper MTX use and monitoring is critical. Finally, identifying factors associated with MTX nonadherence (younger age, male sex, shorter duration of MTX therapy, taking two or more other RA prescription medications) suggests it may be beneficial to develop strategies to help patients remember to take their MTX. Results also suggest that reducing treatment burden (e.g., number of RA prescriptions) without sacrificing efficacy may be a strategy worth evaluating in future research.

## Electronic supplementary material

Below is the link to the electronic supplementary material.

Supplementary material 1 (PDF 188 kb)
