# Supplementary material for: Assessing Methotrexate Adherence in Rheumatoid Arthritis: A Cross-Sectional Survey
Source: Rheumatol Ther. 2015 May 13;2(1):73–84. doi: 10.1007/s40744-015-0011-1 (PMC4883252; doi:10.1007/s40744-015-0011-1)
Supplement: Supplementary file 1 — Supplementary material 1 (PDF 188 kb) [file 40744_2015_11_MOESM1_ESM.pdf]

Rheumatology and Therapy

OPEN ACCESS

PEER REVIEWED SUMMARY SLIDE

- Limited research has been conducted on specific reasons for nonadherence to methotrexate (MTX).
- A sizable percentage of the MTX participants reported nonadherence, most commonly because they forgot, felt it was not needed when feeling well, or had concerns about the long-term safety of MTX.
- Factors associated with MTX nonadherence include younger age, male sex, shorter duration of MTX, and use of ≥two other prescription medications for rheumatoid arthritis (RA).
- Developing strategies for helping patients remember to take medication may be beneficial in increasing overall adherence rates.
- Reducing treatment burden (e.g., number of RA prescriptions) without sacrificing efficacy may be a strategy worth evaluating in future research.

This summary slide represents the opinions of the authors. Support for this study and the associated article processing charges were provided by Genentech, Inc. (South San Francisco, CA, USA). For a full list of acknowledgments and conflicts of interest for all authors of this article, please see the full text online. Copyright © The Author(s) 2015. Creative Commons Attribution Noncommercial License (CC BY-NC).
